# The Bacterial Pathogen *Xylella fastidiosa* Affects the Leaf Ionome of Plant Hosts during Infection

**DOI:** 10.1371/journal.pone.0062945

**Published:** 2013-05-07

**Authors:** Leonardo De La Fuente, Jennifer K. Parker, Jonathan E. Oliver, Shea Granger, Phillip M. Brannen, Edzard van Santen, Paul A. Cobine

**Affiliations:** 1 Department of Entomology and Plant Pathology, Auburn University, Auburn, Alabama, United States of America; 2 Department of Biological Sciences, Auburn University, Auburn, Alabama, United States of America; 3 Department of Plant Pathology, University of Georgia, Athens, Georgia, United States of America; 4 Department of Agronomy and Soils, Auburn University, Auburn, Alabama, United States of America; University of Wisconsin-Milwaukee, United States of America

## Abstract

*Xylella fastidiosa* is a plant pathogenic bacterium that lives inside the host xylem vessels, where it forms biofilm believed to be responsible for disrupting the passage of water and nutrients. Here, *Nicotiana tabacum* was infected with *X. fastidiosa*, and the spatial and temporal changes in the whole-leaf ionome (i.e. the mineral and trace element composition) were measured as the host plant transitioned from healthy to diseased physiological status. The elemental composition of leaves was used as an indicator of the physiological changes in the host at a specific time and relative position during plant development. Bacterial infection was found to cause significant increases in concentrations of calcium prior to the appearance of symptoms and decreases in concentrations of phosphorous after symptoms appeared. Field-collected leaves from multiple varieties of grape, blueberry, and pecan plants grown in different locations over a four-year period in the Southeastern US showed the same alterations in Ca and P. This descriptive ionomics approach characterizes the existence of a mineral element-based response to *X. fastidiosa* using a model system suitable for further manipulation to uncover additional details of the role of mineral elements during plant-pathogen interactions. This is the first report on the dynamics of changes in the ionome of the host plant throughout the process of infection by a pathogen.

## Introduction

*Xylella fastidiosa* is a plant-pathogenic bacterium known to infect several economically important crops, mainly in warm climate regions of the Americas. Host plants affected include citrus, grape, almond, plum, peach, oak, coffee [1], [2], and most recently blueberry [3]. Among the most important and well-characterized diseases caused by *X. fastidiosa* are Pierce’s disease (PD) of grapevine and citrus variegated chlorosis (CVC) of citrus. Symptomatology varies among crops and may include marginal leaf necrosis and scorch (grape, plum, coffee, almond, oak, blueberry), chlorosis (citrus, plum), and/or dwarfing (peach, alfalfa) [2].

*X. fastidiosa* is only found in the water-conducting xylem vessels of plants and in the foregut of different types of xylem-sap feeding leafhopper insect vectors [4]. Though the mechanisms by which the bacterium causes disease are not fully understood, the most plausible explanation is that xylem vessels are colonized and biofilm-like aggregates are formed that disturb the passage of water and nutrients [5]–[8]. However, some researchers argue that water supply to aerial parts of the plants may not be compromised during *X. fastidiosa* infection [9]–11. Whether or not the amount of fluid reaching upper leaves in infected plants is comparable to the amount reaching equivalent leaves in healthy plants, the composition may still differ in either case, affecting the nutritional status of infected leaves. Here, using a model host, we tested the hypothesis that the presence of *X. fastidiosa* causes changes in the mineral status of the host plant that may contribute to successful infection and symptom development. To test this hypothesis, the spatial and temporal dynamics of the plant host leaf ionome, viz. “the mineral nutrient and trace elements found in an organism” [12], during infection were characterized.

Understanding the dynamics of the plant host ionome during infection allows for the possibility of finding novel molecular components involved in the plant-pathogen interaction. Ionomics is an approach to obtain a snapshot of information about the functional state of an organism under certain conditions at a specific time [13]–[16]. Previous work has established that studying the ionome of leaves is an appropriate indicator of the physiological status of the whole plant [16]; therefore, leaves were used as the focal point of this work to elucidate the effects of bacterial infection on the host. Examination of the ionome of leaves is especially relevant for a xylem-limited bacterium such as *X. fastidiosa*, since mineral nutrients necessary for plant growth and reproduction are obtained from the soil via roots, distributed within the plant through its vascular system, and ultimately deposited in plant leaves.

Essential micro- and macronutrients are required for a myriad of functions within cells [17], [18] and are under tight homeostatic control because, although required, many are also toxic at high concentrations. The mineral elements required for survival of bacteria are essentially the same as those required for plants [19]. Therefore, bacteria have evolved strategies to bypass host defenses and ‘outcompete’ the host for certain elements, while hosts have developed ways to limit pathogens by depriving them of or intoxicating them with specific elements. In the 1970s, the term ‘nutritional immunity’ [20] was coined to refer to a control strategy against microbial invasion exhibited by animal hosts who deprive bacterial pathogens of Fe, and later was expanded to describe competition for other elements [21]. Understanding nutritional immunity in plants could open possibilities for the development of new disease control methods using specific chelators and inhibitors of mineral transport, analogous to the manipulation of metal elements used in animals against bacterial infection [21], [22]. By studying the dynamics of the leaf ionome during bacterial infection, we sought to discover mineral element manipulations induced by *X. fastidiosa* in order to uncover an underlying nutritional immunity process during this plant-bacterium interaction.

Previous studies of *X. fastidiosa* suggest that divalent cations (Ca, Mg) can have non-specific effects on colonization by facilitating adhesion between negatively-charged bacterial cells and xylem vessels [23]. Additionally, Ca specifically regulates virulence traits in *X. fastidiosa* including increased biofilm formation, adhesion to surfaces and other cells, and twitching motility *in vitro* [24]. In fact, cells from the pathogenic biofilm state of *X. fastidiosa* have been shown to significantly accumulate particular mineral elements (Ca, Cu, K, Mn, and Zn) as compared to planktonic cells [25]. According to gene expression studies, Fe promotes virulence factors such as type IV pili and bacteriocins [26]. Concentrations of Cu and Zn present in xylem fluid have been correlated to *in vitro* growth of *X. fastidiosa* [27]. Moreover, a *X. fastidiosa* Zn-protease is induced in citrus to utilize free amino acids in the xylem as nitrogen and carbon sources [28]. These studies suggest that the mineral content of the host plant could impact the virulence of this bacterium. Additionally, previous studies of limited scope have suggested that *X. fastidiosa* infection affects the mineral nutrition status of plant hosts in the field [11], [29], [30].

The objective of the present study was to understand the effects of *X. fastidiosa* infection on the host plant by using the plant leaf ionome as an indicator of plant physiological status to test the hypothesis that infection leads to an ionomic imbalance that may affect disease development. Dynamic changes in the ionome of the model host plant, *Nicotiana tabacum*, were characterized spatially and temporally during growth in the greenhouse with and without infection by *X. fastidiosa*. Findings from the greenhouse study were correlated to corresponding changes in infected natural hosts in the field, including multiple varieties of grape, blueberry, and pecan. Results show that *X. fastidiosa* infection causes significant changes in the levels of specific mineral elements in the host plant, and, together with results from others [11], [29], [30], suggest that specific mineral changes may be a pervasive effect of *X. fastidiosa* infection in plant hosts.

## Materials and Methods

### Greenhouse Experiments

Tobacco plants were propagated in the greenhouse and inoculated with *X. fastidiosa* according to a protocol previously described [31]. *Nicotiana tabacum* ‘Petite Havana SR1’ seeds (Plant Introduction (PI) number 552516) were obtained from the USDA Germplasm Resources Information Network (GRIN) and germinated in Sunshine® Mix #8 (Sun Gro Horticulture Canada Ltd., Vancouver, Canada). Greenhouse temperature was maintained between ∼20–25°C and natural sunlight was used. After ∼1 month, 50 seedlings were transplanted into 4.5″ square pots. From this point onward, plants were fertilized occasionally with Peters Professional® 20-10-20 Peat-Lite Special® (The Scotts Company, Marysville, OH), only when yellowing of leaves (deficiency) was apparent (∼three times during sampling period).

At ∼1 month post-transplant, tobacco plants were prepared for inoculation by cutting the top of the stem and removing bottom juvenile leaves so that only three healthy adult leaves in the lower portion of the plant remained (numbered 1–3). This time point was designated time zero and two leaf samples of excised tissue were collected from each of five random plants (one leaf collected from upper removed leaves and one leaf collected from the bottom removed leaves). Bacterial inoculum was prepared from *X. fastidiosa* type strain Temecula [32] cultured on PW solid media [33] at 28°C for ∼1 week. Bacteria from two plates were scraped off and resuspended in 1.5 ml succinate-citrate phosphate buffer [34]. A 1 ml tuberculin syringe with a 23 gauge needle was used to inject half of the plants with ∼20 µl inoculum per each remaining tobacco petiole, near the axils. The other half of the tobacco plants (control plants) were injected in the same manner with buffer only.

Plants continued growing from the site where the stem was cut (leaves numbered 4–10). After ∼3 weeks – designated time point 1– all leaves were sampled from ten random plants (five inoculated and five control). Leaves were classified according to their appearance as control (healthy) or senescent (showing browning symptoms) from buffer-inoculated control plants and asymptomatic (healthy) or symptomatic (marginal leaf scorch) from *X. fastidiosa*-inoculated plants. All leaves from ten additional plants (five inoculated and five control) were harvested approximately every seven to ten days in the same manner at time points 2, 3, 4, and 5. Greenhouse experiments were repeated three times independently. Specific sampling times were as follows: 18, 27, 39, 48, and 59 days post-inoculation (dpi) in experiment 1; 25, 34, 40, 47, and 56 dpi in experiment 2; and 20, 28, 34, 41, and 49 dpi in experiment 3.

### Ionome Characterization

Leaves were prepared for analyses by drying at 65°C for 1 hour. To avoid sampling biases from ‘hole punch’ sampling, whole leaves were sampled for ionome analyses. The leaves were then crushed to a fine powder by mortar and pestle and sampled at 5 and 10 mg of dry weight. Samples were digested for at least 1 hour at 100°C in 200 µL of mineral-free concentrated nitric acid (OPTIMA, Fisher Scientific). After dilution with ultra-pure, mineral-free water and centrifugation at 13,000×*g* to remove any remaining particulates, samples were analyzed by inductively coupled plasma with optical emission spectroscopy (ICP-OES, Perkin Elmer 7100 DV, Waltham, MA) with simultaneous measurement of Ca, Co, Cu, Fe, K, Mg, Mn, Mo, Na, P, S, and Zn. Mineral concentrations were determined by comparing emission intensities to a standard curve created from certified mineral standards (SPEX CertiPrep, Metuchen, NJ). Standard curves were confirmed by re-analysis of standard solutions diluted in a matrix equivalent to the sample. Individual readings are the average of two intensity measurements and varied by less than 5%. Repeated analysis of different amounts (5 and 10 mg) of individual samples showed less than 5% variation.

### Quantification of Bacterial Populations

*Xylella fastidiosa* bacterial populations in tobacco leaves were quantified via quantitative polymerase chain reaction (qPCR) using the previously described HL5/HL6/HLP primer and TaqMan™ probe set [35]. A portion of the lower petiole (∼3 cm×1 cm; ∼100–300 mg) of each leaf was excised, and the weight of tissue recorded. DNA from each tissue sample was extracted using a modified CTAB protocol [36] and resuspended in 500 µl molecular grade water. DNA was amplified on an Applied Biosystems (ABI) 7500 Real-Time PCR System (Life Technologies Corporation, Carlsbad, CA) in reactions (25 µl) containing the following components: 1X ABsolute™ Blue QPCR ROX Mix (ABgene® UK, Epsom, Surrey), 0.2 µM each primer, 0.4 µM probe (labeled 5′-6FAM, 3′-BHQ1), and 1 µl DNA template. Cycling parameters were 95°C for 15 min, followed by 40 cycles of 95°C for 15 s and 60°C for 1 min.

A four-point standard curve was amplified alongside the tobacco samples in each qPCR for quantification of the unknowns. Standards were created from 10-fold serial dilutions of DNA extracted from a known quantity of *X. fastidiosa* colonies. *X. fastidiosa* colonies were enumerated by spread-plating serial dilutions of the same cell suspension used for DNA extraction on PW solid media [33] and counting colony forming units (CFU) visible after two weeks of incubation at 28°C. Finally, interpolated qPCR CFU values of the unknowns were divided by the previously recorded weight of leaf tissue extracted to determine *X. fastidiosa* CFU/mg leaf tissue for each leaf.

### Field Sample Collection

Samples of symptomatic leaves from plants appearing to be naturally infected with *X. fastidiosa* as well as leaves from control asymptomatic plants from the same sample site were collected from commercial fields in Alabama (AL) and Georgia (GA) from 2009–2012 (Table 1). Field access to privately owned land was granted by each owner. Samples from grape (*Vitis* sp.) were collected from four sites in Lumpkin County (GA) in October 2009, September 2010, and October 2012. Samples from blueberry (*Vaccinium* sp.) were collected from three sites in Alma, Clinch, and Pierce Counties (GA) in September 2011 and 2012. Samples from pecan (*Carya illinoinensis*) were collected from one site in Lowndes County (AL) in August 2010 and September 2011 and from Tift County (GA) in September 2012. Leaf samples were transported at 4°C and stored at −20°C until analysis by PCR and ICP-OES was performed. The petiole was removed from a single leaf from each sample and DNA was extracted as described previously. The resulting DNA was PCR-amplified to confirm the presence of *X. fastidiosa* using the previously described RST31/RST33 primer set [34] and gel electrophoresis of the amplification product, or analyzed by qPCR as described above. The remaining portion of the same leaf was used for ICP-OES. Only samples where *X. fastidiosa* was confirmed by PCR were used to compare to non-infected (PCR negative, asymptomatic) leaves from the same or neighboring plants. In total, 33 pairs of grape samples, 9 pairs of blueberry samples, and 13 pairs of pecan samples were considered.

**Table 1:** Changes in element concentration of field-grown host plants after infection with *Xylella fastidiosa.*

Host Plant	State	Year	n^1^	% change from control^2^	
				Ca	P
Grapevine (*Vitis* sp.)					
*V. vinifera* ‘Cabernet Sauvignon’	GA	2009	2	31±6	ND^3^
*V. vinifera* ‘Cabernet Sauvignon’	GA	2010	5	96±30	−27±8
*V. vinifera* ‘Cabernet Sauvignon’	GA	2012	3	−29±28	−9±13
*V. vinifera* ‘Chardonnay’	GA	2010	2	152±55	8±2
*V. vinifera* ‘Chardonnay’	GA	2012	2	149±46	−33±8
*V. vinifera* ‘Merlot ‘	GA	2009	2	90±19	ND
*V. vinifera* ‘Merlot’	GA	2010	4	30±6	−23±7
*V. vinifera* ‘Merlot’	GA	2012	1	90	−14
*V. vinifera* ‘Mourvedre’	GA	2009	4	45±9	ND
*V. vinifera* ‘Petit Manseng’	GA	2010	3	145±15	−18±2
*V. vinifera* ‘Touriga’	GA	2012	2	−28±29	−36±18
*V.* ‘Vidal’	GA	2009	1	43	ND
*V.* ‘Vidal Blanc’	GA	2012	2	−34±14	8±30
Blueberry (*Vaccinium* sp.)					
‘Star’	GA	2011	2	170±75	5±1
‘Star’	GA	2012	5	76±20	10±6
‘FL 86-19’	GA	2011	2	28±9	2±1
Pecan (*Carya illinoinensis*)					
‘Cape Fear’	AL	2010	2	36±9	−12±2
‘Cape Fear’	AL	2011	1	13	5
‘Cape Fear’	AL	2012	10	−6±7	−8±6

1 n = number of pairs analyzed for each plant species. A pair consisted of a non-infected asymptomatic leaf and a symptomatic leaf infected (confirmed by *X. fastidiosa*-specific PCR) taken from the same or a neighboring plant.

2 Metal content determined by ICP-OES. Data represents average and standard error of percentage of changes in elemental composition of infected vs. non-infected leaves.

3 ND = not determined.

### Statistical Analysis

Canonical discriminant analysis (CDA) as implemented in SAS® PROC CANDSIC was used initially to assess the joint importance of the total ionome in the discrimination among classes, where class was defined as the combination of treatment (infected and non-infected with *X. fastidiosa*) and leaf position (#1–8). The CDA was repeated using only positions #4–8. For the CDAs, two sets of the experimental data were used because they represented a balanced data set with no missing observations.

For the analyses of individual mineral concentrations, mixed models methodology was used as implemented in SAS® PROC MIXED. Treatment was defined as a leaf infected or non-infected with *X. fastidiosa* (as established by qPCR analysis). Position, relative sampling time, and their interactions were considered main effects. Experimental repetition (set) and plant (set*treatment*time) were random effects. The probability for pairwise differences was assessed using the simulation option of the LSMEANS command to guard against an inflated Type I error.

The frequency of paired field samples conforming to the results from our greenhouse study (increased concentrations of Ca and decreased concentrations of P) was assessed by comparing the observed frequency of results with the expected frequency using the binomial distribution (*p*<0.05) in Microsoft Excel® 2010. For the analysis of leaf position, one-way ANOVA (or Kruskal Wallis for non-normal data) was used and means separated by Fisher’s protected LSD (*p*<0.05) using Statistix 8.0 (Analytical Software, St. Paul, MN).

## Results

### Assessment of the *X. fastidiosa-*tobacco System for Ionomic Studies

**Figure 1:**
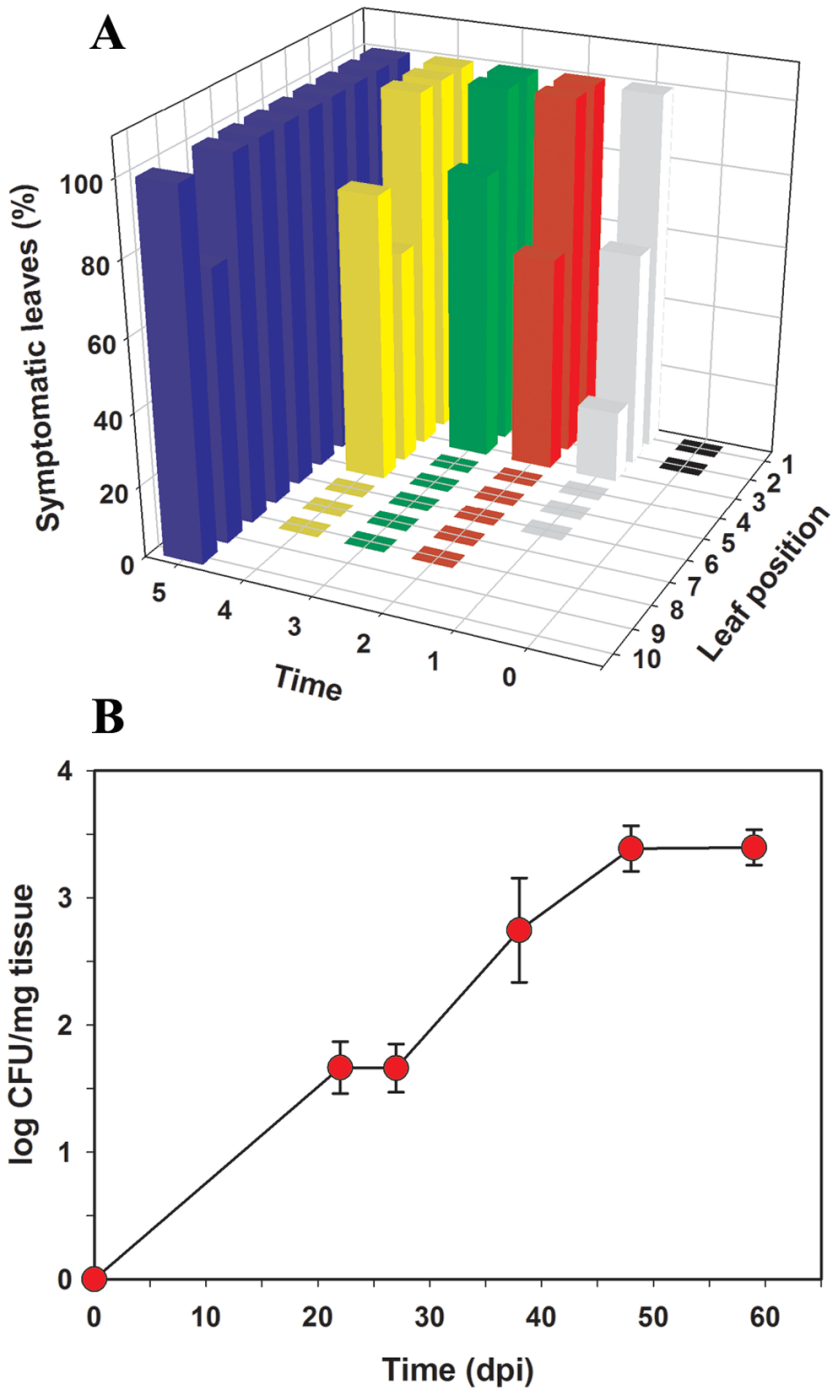
Infection of *Nicotiana tabacum* ‘Petite Havana SR1’ with *Xylella fastidiosa* **.** A) Progression of symptom development in tobacco plants infected with *X. fastidiosa* Temecula. The percentage of symptomatic leaves showing marginal leaf scorch at a specific leaf position on the plant (increasing numbers from bottom to top of the plant) and at a specific time are represented in the graph. Sampling times numbered 1 through 5 in the graph correspond to 0, 22, 27, 38, 48, and 59 days post inoculation, respectively. Five tobacco plants were analyzed at each sampling time. The experiment was repeated three times and data in the graph corresponds to one representative experiment. B) Population of *X. fastidiosa* Temecula in petioles of infected tobacco plants. Bacterial populations were quantified by specific qPCR using sections of petioles as source material. Five tobacco plants were analyzed per sampling time at several days post-infection (dpi). Data represented in the graph correspond to means and standard errors of bacterial populations of all leaves at positions ≥#4 at each sampling time. The experiment was repeated three times and data in the graph corresponds to one representative experiment.

To facilitate ionomic studies in plants infected with *X. fastidiosa*, a previously described model system, *Nicotiana tabacum* ‘Petite Havana SR1’ (tobacco), was used [31]. Relative position on the tobacco plants and visual symptom appearance (marginal leaf scorch) were recorded for each leaf. Symptoms were detected in leaves at position #4 and above approximately 41–48 days post inoculation (dpi) (Fig. 1), a typical latency period previously observed [1], [31], [37]. Symptoms in leaves from the bottom three positions (#1–3) were detected earlier, but, due to significant contributions from natural senescence and the fact that these leaves were directly needle-inoculated, these symptoms were not considered diagnostic of successful infection. Once symptoms were detected in any of the leaves at positions #4 and above, they rapidly (ca. 8–12 days) spread to the rest of the leaves acropetally, as is typical for *X. fastidiosa* infections [31], [38]. Populations of the bacterium in leaves #4 and above increased rapidly from 25–30 dpi, indicating movement of the bacterium inside xylem vessels, and reached a plateau (ca. log 3.4 CFU/mg tissue) after approximately 50 dpi (Fig. 1).

### Multivariate Analysis of Tobacco Greenhouse Experiments

**Figure 2:**
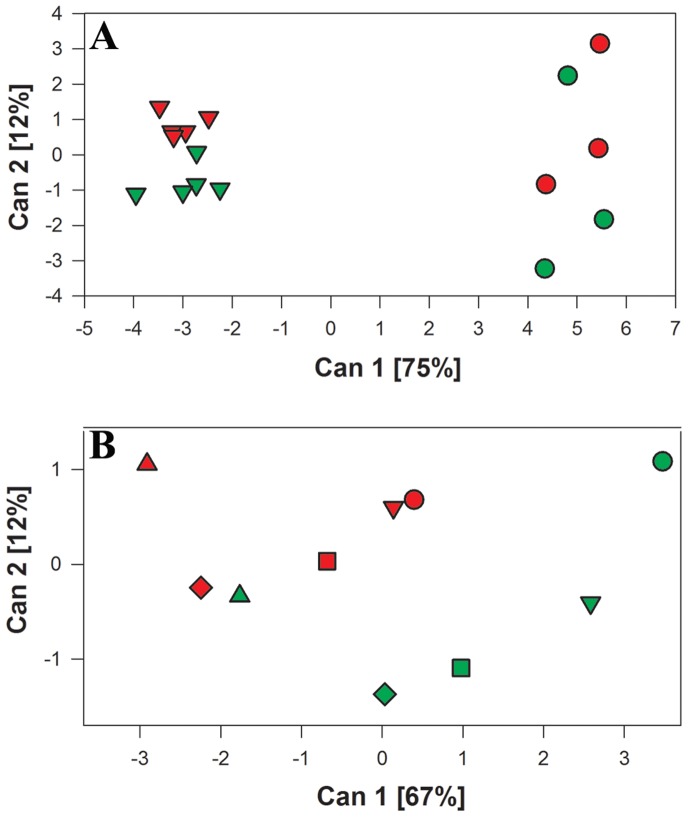
Canonical discriminate analysis of treatment and leaf position as classification variables. Concentrations of 12 elements analyzed were considered as independent variables, while experimental set and time points were considered as replicates. A) Leaves were separated according to relative position in the plant between leaves #1–3 (circles**)** which are older, directly-inoculated and leaves ≥ #4 (inverted triangles) that represent new growth after inoculation. B) Leaves at the same position were compared according to presence or absence of *X. fastidiosa*. Circle = position #4; inverted triangle** = **position #5; square** = **position #6; diamond** = **position #7; triangle** = **position #8. Phenotypic correlations for Can1 are driven significantly (*p*<0.05) by Ca (r = −0.98), Mg (r = −0.89), Na (r = 0.85), K (r = 0.74), and Mn (r = −0.59) and for Can2 by Cu (r = 0.57) and S (r = −0.52). B, Fe, Mo, P, and Zn have no significant effect on discrimination among classes. Red symbols indicate leaves infected with *X. fastidiosa* and green symbols indicate non-infected leaves. The length of the axes is proportional to the accounted for percentage of the total multivariance.

Initially, multivariate statistical analysis was conducted to establish the joint effect of all variables considered. Sampled leaves were classified by treatment according to the following: i) “infected”, including all leaves from bacterium-inoculated plants that were positive for *X. fastidiosa* by qPCR; and ii) “non-infected”, including all leaves from buffer-inoculated plants and leaves from bacterium-inoculated plants that were negative for *X. fastidiosa* by qPCR. This classification was used throughout the analysis, as having detectable levels of *X. fastidiosa* was considered the best indication of the infection status of a particular leaf at a specific time. Canonical discriminant analysis (CDA) was performed considering treatment and leaf position as classification variables, while time and experimental set were considered as replication variables. The concentrations of the 12 elements (B, Ca, Cu, Fe, K, Mg, Mo, Mn, Na, P, S, and Zn) were considered as independent variables (Fig. 2A). Leaves were grouped according to their position in the plant: leaves from positions #1–3 (older leaves, needle-inoculated) or leaves from positions ≥ #4 (new growth after inoculation). Graphing centroid means for canonical variate 2 (Can2, 12% of the variation) against canonical variate 1 (Can1, 75% of the variation) demonstrates that leaves in positions #1–3 were fundamentally different from the ones in positions ≥ #4. Leaves with position ≥ #4 clustered together according to treatment (Fig. 2A, green and red inverted triangles) along Can2. This indicates that a treatment effect could be dissected among leaves ≥ #4 that were colonized by *X. fastidiosa* during the course of the experiment; therefore, these leaves were used for further analyses.

Focusing on newly grown leaves that develop downstream from the inoculation site provides better insight into the dynamics of disease development. These upper leaves (≥ #4) were not infected directly through needle inoculation, but rather became infected by bacterial movement through the host, therefore avoiding experimental biases due to excessive local inoculum delivery. Moreover, as mentioned above, leaves #1–3, by being older, undergo natural senescence earlier than bacterial infection symptoms can be observed, so they were excluded from further analyses. For the subsequent CDA, leaf position and treatment were considered as variables, whereas time and experimental set served as replicate observations. In this analysis, Can1 explained 67% of the variation, while Can2 explained 12% (Fig. 2B). A tendency was observed for each leaf position (#4 through 8) where non-infected leaves had a more positive Can1 mean and separated with similar distances from leaves at the corresponding position infected with the bacterium (compare green and red symbols, non-infected and infected, respectively, in Fig. 2B). With the exception of leaf #8 (Fig. 2B, green and red triangles), separate groups can be resolved between non-infected and infected leaves. The exception with leaf #8 (positioned at the top of the plant) may be explained by reduced numbers of samples due to the fact that some plants had 7 leaves or less at earlier sampling times. Phenotypic correlations showed that differences along Can1 are driven significantly (*p*<0.05) by Ca, Mg, Na, K, and Mn (r = −0.98, −0.89, 0.85, 0.74, and −0.59, respectively) and for Can2 by Cu (r = 0.57) and S (r = −0.52). B, Fe, Mo, P, and Zn have much smaller effects on discrimination among classes. To dissect these differences, each element was considered individually.

### Overall Changes in Total Ionome of Tobacco Infected with *X. fastidiosa*

**Figure 3:**
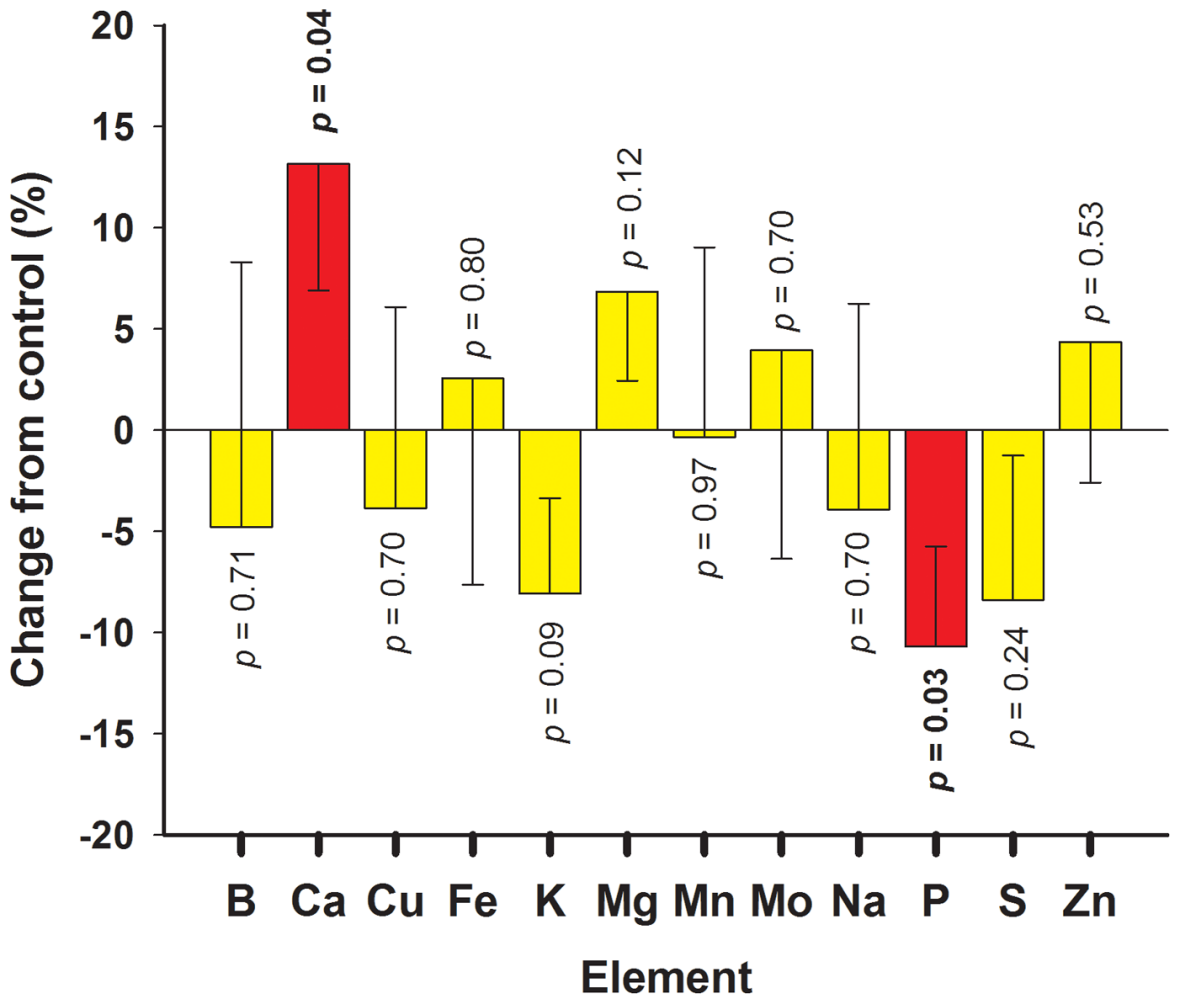
Changes in total ionome of tobacco leaves infected with *Xylella fastidiosa*. Tobacco plants (*Nicotiana tabacum* ‘Petite Havana SR1’) growing in the greenhouse were inoculated with *X. fastidiosa* or buffer (control) and the ionome of each leaf was characterized by inductively coupled plasma optical emission spectroscopy (ICP-OES). Mean values of element concentrations (in mg/g of plant tissue) were obtained from leaves in positions ≥ #4. Mean relative percentage of change with corresponding standard errors (represented only towards 0 value) were calculated by comparing mean leaf concentrations across five time points and three experimental sets between leaves where *X. fastidiosa* was detected (‘infected’) against those were the bacterium was not detected (‘non-infected’) (n∼375/treatment). *P* values for main treatment effects are included for each element; those highlighted in bold with red bars are considered significant (*p*<0.05).

For an overall assessment of the effect of infection on the ionome of tobacco leaves when *X. fastidiosa* is present, all leaves in positions ≥ #4 collected during five sampling time points in three experimental sets were considered (Fig. 3). The overall treatment*time*position (*p*≥0.60) and treatment*position (*p*≥0.32) interactions were not significant sources of variation in these experiments. Based on this, further analysis focused on the effect of the treatment (infected vs. non-infected leaves) on the concentrations of elements analyzed. Significant treatment effects were observed for Ca (*p* = 0.04, DF = 118) and P (*p* = 0.03, DF = 122) when a *p* = 0.05 threshold was considered. Considering overall means from all leaves collected over five time points during three experimental sets, Ca concentration increased by 13% (Fig. 3) in leaves with *X. fastidiosa* compared to leaves without the bacterium (18.8 to 21.3 mg/g tissue). P was reduced by 11% in leaves with *X. fastidiosa* (3.7 to 3.3 mg/g tissue) (Fig. 3). Changes in the concentration of other elements between infected and non-infected leaves were non-significant (*p*>0.05). This analysis served to identify elements of interest (Ca and P) but do not reflect changes in element concentrations at a specific time or leaf location, which are considered below and provide a better representation of the dynamics of disease progression.

### Temporal Dynamics of Mineral Element Levels during Disease Progression

The relationship between time after infection and changes in the plant host ionome was studied. Time had a significant effect on the concentration of Ca (*p*<0.0001), B, Cu, K, Mg, Mn, Mo, Na, and P (*p*<0.03) but no effect in the concentration of Fe, S, and Zn (*p*>0.05). When comparing regression analyses of ionomic changes over time in infected vs. non-infected leaves, the only difference in slope trending towards significance was with Ca (*p* = 0.08). The total increase in Ca concentration in infected tobacco leaves (y = 10.8+3.1×, *p*<0.001) occurred more rapidly than in non-infected leaves (y = 13.3+1.7×, *p* = 0.003), as shown by the respective regression slopes. Additionally, in infected plants, Ca concentrations were higher at most time points during the course of infection, and the difference among infected vs. non-infected leaves was exaggerated after time point 3 (34–40 dpi), representing an overall ∼30% (23.0 to 17.6 mg/g tissue) increase at this time point when all leaves and experimental repetitions were considered. Time point 3 samples were taken a few days before symptoms were evident in leaves ≥ #4 (time 4, 41–48 dpi) (Fig. 1). By time point 5, when symptoms were evident in most leaves, Ca in infected leaves was ∼27% higher than in non-infected leaves (27.4 to 21.6 mg/g tissue). No significant regressions (*p*>0.05) could be fit to the data for any of the other elements over time, either for infected or non-infected treatments. No significant (*p*>0.05) differences in the intercepts were found for the other minerals analyzed, indicating that all plants have similar initial mineral concentrations.

### Effect of Leaf Position on Ionomic Changes in Infected Tobacco Leaves

**Figure 4:**
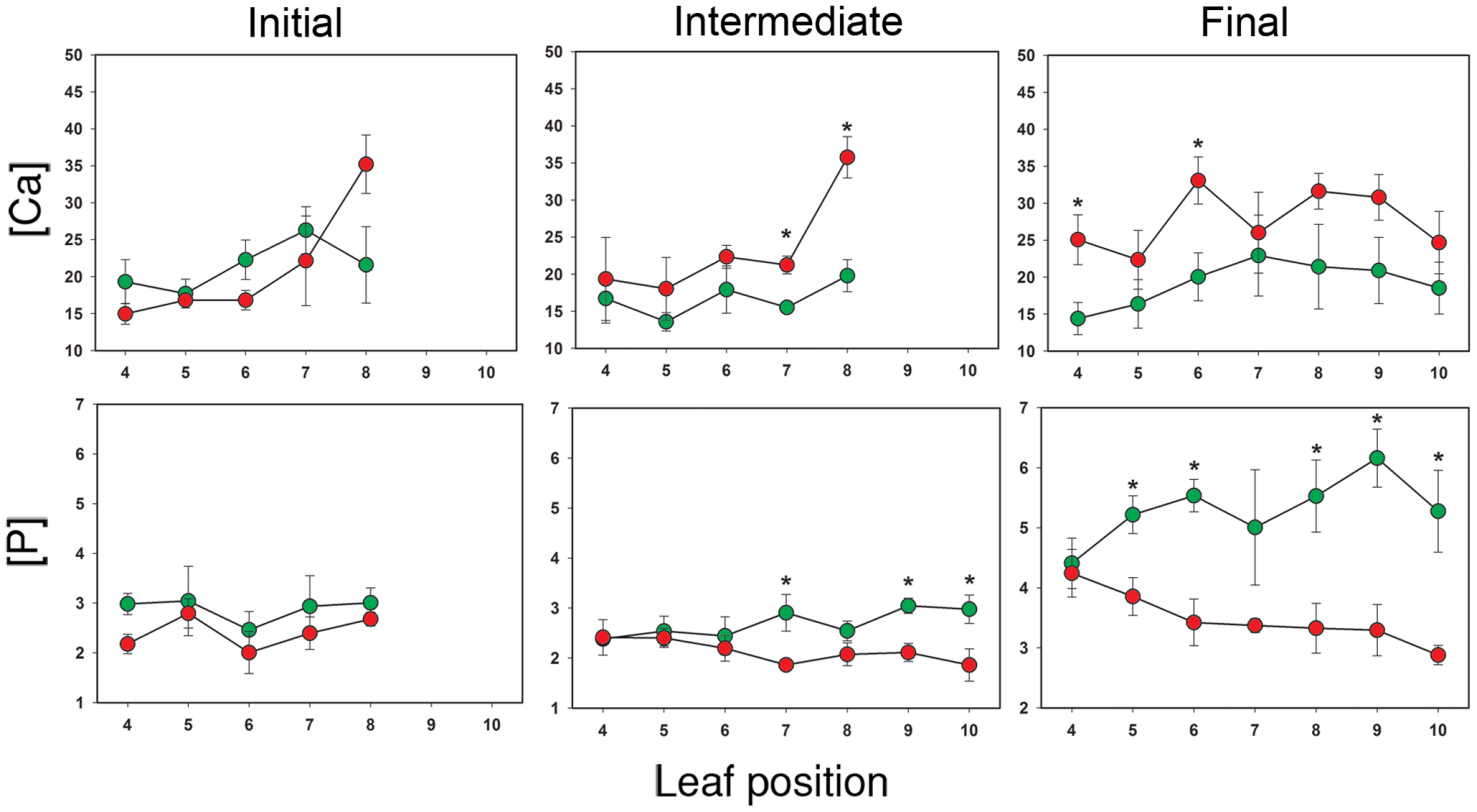
Changes in element concentrations by leaf position over time. Element concentrations in tobacco leaves inoculated with buffer (green circle, control plants) or *X. fastidiosa* cell suspension (red circle, infected treatment) were followed over time considering relative leaf position in the plant (1 = most basal leaf, 10 = most apical leaf). Data correspond to leaves in positions ≥#4. For the first column (initial), leaves were collected between 25–27 days post infection (dpi); for the second column (intermediate), samples were collected 39–47 dpi; and for the third column (final), samples were obtained 56–59 dpi. From top to bottom, rows of graphs correspond to concentrations of Ca and P expressed in mg per g of plant tissue. Values represent means and standard errors (n = 5) from one out of three experimental sets conducted. *Indicates significant difference (*p*<0.05) between treatments at a specific leaf position according to one-way ANOVA or Kruskal-Wallis.

*X. fastidiosa* moves acropetally inside the xylem vessels of the host plant aided by the xylem flow. Leaf position had a significant effect on the concentration of Ca (*p*<0.0001), Cu, K, Mg, Mn, Mo, Na, and Zn (*p*<0.03), but had no effect on the concentration of B, Fe, P and S (*p*>0.05). To assess the influence of relative leaf position during disease development, mean element concentrations were considered for each leaf position. This analysis suggests that differences between treatments are accentuated as time progresses (Fig. 4). Comparisons of leaves in identical leaf positions from plants inoculated with buffer versus those inoculated with the bacterium show that the increase in Ca concentration in infected plants becomes evident in the upper leaves at approximately 39 dpi (“intermediate” column in Fig. 4). This timeframe coincides with exponential growth of *X. fastidiosa* in the plant and precedes visualization of symptoms (Fig. 1). By 59 dpi, differences in Ca were distributed across all leaves (“final” column in Fig. 4). As an example, at leaf position #9 for the experiment represented in Figure 4, infected leaves had ∼47% (30.8 to 20.9 mg/g tissue, *p = *0.11) more Ca than the buffer-inoculated control. Deficiencies in P concentrations in infected leaves were evident towards the end of the experiments when leaves were symptomatic (56 dpi, “final” column Fig. 4). For the experiment represented in Figure 4, leaves at position #9 showed a decrease of ∼46% (3.3 to 6.1 mg/g tissue, *p = *0.02) in concentration of P compared to control plants.

### Changes in Ionomes of Infected Host Plants Collected from the Field

A field survey was conducted to observe the impact of *X. fastidiosa* infection on the ionome of natural plant hosts. Leaf samples from grape, blueberry, and pecan plants naturally infected with *X. fastidiosa* were collected from fields in Alabama and Georgia over four consecutive years. Presence of the bacterium was confirmed by PCR and the ionome characterized by ICP-OES for paired symptomatic/infected and asymptomatic/non-infected leaves from the same or neighboring plant. In Table 2, the mineral element concentrations in leaves of field samples are compared to the tobacco greenhouse values. Data shown was pooled for the different grape (*Vitis* sp.) and blueberry (*Vaccinium* sp.) cultivars. This data shows that: (1) average elemental concentration values vary based on the host; and (2) variations in element concentration tend to be relatively small (SE ∼10%) even when considering that samples from the field were collected over multiple years and across different locations and plant varieties. When comparing infected and non-infected leaves from the field (Table 1), infected leaves had higher Ca and lower P than non-infected leaves in approximately 79 and 60% of paired samples, respectively. A binomial test of the field samples indicates a significant increase in Ca (*p<*0.001) and decrease in P (*p* = 0.027). In fact, a few exceptions notwithstanding, the percent change in elemental compositions during infection was more pronounced in the field than what was observed in the greenhouse with tobacco (Table 1, Figure 3).

**Table 2:** Ionome comparisons of greenhouse tobacco and field-grown host plants.

Host plant		Element concentration (mg/g tissue)^1^									
		Ca	Cu	Fe	K	Mg	Mn	Na	P	S	Zn
*N. tabacum* (greenhouse) n = 450	mean	20.2	0.003	0.04	20.3	5.2	0.03	0.41	2.7	2.9	0.03
	SE	0.4	9E-5	1E-3	0.4	0.08	6E-4	0.02	0.06	0.1	7E-4
*Vitis sp.* (field) n = 83	mean	15.3	0.09	0.07	12.1	2.3	0.26	0.03	3.65	2.22	0.04
	SE	0.6	0.02	2E-3	0.56	0.09	0.02	2E-3	0.25	0.05	3E-3
*Vaccinium sp*. (field) n = 33	mean	5.9	0.004	0.05	3.8	2.2	0.1	0.04	2.9	1.9	0.01
	SE	0.5	3E-4	7E-3	0.22	0.13	0.01	6E-3	0.09	0.08	7E-4
*Carya illinoinensis* (field) n = 34	mean	16.9	0.007	0.04	8.3	2.2	0.6	0.02	2.9	1.9	0.1
	SE	1.3	2E-3	1E-3	0.41	0.12	0.05	2E-3	0.14	0.08	9E-3

1 Mean and standard error (SE) are calculated from all infected and non-infected leaves at all positions in greenhouse experiments.

## Discussion

The ionomic approach presented here is the first spatial and temporal analysis of host mineral content response to infection by a xylem-limited bacterium. *X. fastidiosa* has no type III secretion system [39] and lives inside xylem vessels which consist largely of non-living tissue; nevertheless, infection induces plant defense responses such as tylose and gum formation [40], induction of pathogenicity-related (PR) genes [41], and production of phenolic compounds [42]. The question of how the plant host senses the presence of the bacterium remains unanswered, although recently it has been suggested that the living tissue at the xylem periphery may sense and respond to infection by *X. fastidiosa* [41]. Future studies are needed to determine the exact mechanisms of how the presence of the bacterium induces the increase in Ca and decrease in P in the host.

Previous studies have indicated that the mineral content of *X. fastidiosa*-infected plants differs from non-infected ones in hosts such as grape [29], citrus [30], and peach [11], while one report in peach found no differences [43]. Those studies were conducted with single time point field samples from one variety of grape (*Vitis vinifera* ‘Chardonnay’) [29] grown in California (USA), one of peach (*Prunus persica* ‘Flordaking’) grown in Florida (USA) [11], and one of citrus (*Citrus sinensis* ‘Pera’) grown in São Paulo (Brazil) [30]. The present study augments the field observations from previous work to include eight varieties of grape (four sites), two varieties of blueberry (three sites), and one pecan variety (two sites) grown in Georgia and Alabama, sampled in four different years. The significant modifications of Ca and P during *X. fastidiosa* infection found in the present study, both in the greenhouse (tobacco) and the field (blueberry, pecan, grape), were in agreement with the significant increases in Ca in grape [29] and the reductions in P in citrus and peach [11], [30] previously reported. The present study greatly expands upon previous findings by indicating the pervasive nature of these effects and observing these tendencies in hosts and environmental conditions not previously tested. Furthermore, none of the previous studies, perhaps due to the inherent variability of field observations, detected significant changes in Ca and P concurrently, as in the present study. The present study also shows that these significant changes occur in both symptomatic and asymptomatic tissue, which has not previously been demonstrated. In addition, our multivariate analysis reveals a complex interaction between different elements. Canonical separations among infected and non-infected leaves were not fully resolved into discrete groups, perhaps due to insufficient replication and the variability of these experiments; however, the main goal of this analysis was to show that mineral elements were significantly modified by bacterial infection.

The main ionomic effects observed in this study were in macronutrients, while changes in essential micronutrients may not have been detected due to much tighter homeostatic ranges for these elements, i.e., small changes could lead to more significant physiological changes. This could also be attributed to the greenhouse experimental design, where tobacco growth at different times of the year introduced variability that could further obscure changes in micronutrients. Moreover, it was assumed that replicate plants are at a similar stage of disease progression, justifying destructive sampling of a subset of plants at each time point; however, this assumption cannot be verified due to the terminal nature of the analysis. The observed ionomic changes are believed to be due to redistribution of minerals by the host plant in response to infection and do not reflect bacterial ion content since the contribution of bacterial biomass to the leaf samples is negligible.

The strongest effect of infection was found with Ca, the concentration of which increased in upper leaves of infected plants prior to symptom development. Calcium serves several functions in the plant, including contributing to the rigidity of cell membranes and primary cell walls and interacting with phytohormones involved in cell elongation [44]. Increases in cytoplasmic calcium concentrations occur in plants as a response to multiple signals, such as biotic and abiotic stresses, and have been related to the defense response of the host plant [45]. The experimental design used here cannot determine if the increase in Ca concentration in leaves corresponds to an increase in cytoplasmic or apoplastic Ca. Calcium in plants is taken up from the soil at the root tip and is later loaded into the xylem conduits by specific transporters [46], [47]. Once Ca reaches the leaves, it is no longer mobile [48], so the increases in leaf Ca detected here may correspond to higher uptake from the roots and transit through the xylem. *Arabidopsis thaliana* responding to biotic or abiotic stresses was shown to increase Ca at the whole plant level [49]. Interestingly, a recent publication showed that grapevines respond to *X. fastidiosa* infection and water stress by up-regulating genes involved in Ca-signaling [41], which may be related to the observations reported here. Our research group [24] showed that Ca concentration in culture media *in vitro* modulates *X. fastidiosa* attachment to surfaces and other cells, biofilm levels, and twitching motility, and is significantly accumulated in biofilm cells [25]. Previous studies showed that *X. fastidiosa*-occluded xylem vessels had higher concentrations of Ca compared to non-occluded vessels [23], and Ca has also recently been linked to sieve-tube occlusion by phytoplasmas, another vascular pathogen [50]. It remains to be determined if the increases in Ca leaf concentrations observed here correlate with increased Ca movement through the xylem, which could benefit *X. fastidiosa* infection by augmenting biofilm formation and movement. Further studies, including measurements of Ca in xylem sap from different parts of the plant, are needed to confirm this but are complicated by temporal variability of mineral element concentrations in xylem sap [51].

Reductions in P were observed in infected tobacco leaves in the greenhouse and in grape and pecan samples from the field, but not in blueberry samples from the field. This may indicate that this effect is less pronounced in blueberry and/or these results are biased by the small sample size. Phosphorus is important to plants for energy storage and transfer as part of ADP and ATP. Due to the accumulation of anthocyanin, deficiencies in P in some plant hosts can change the color of stems and leaves to dark green, red, or purple [52], [53], which resembles the symptoms caused by *X. fastidiosa* in grape and blueberry [2]. However, more research is needed to correlate P deficiencies with symptomatology. The influence of *X. fastidiosa* on reduced plant availability of P could be explained by: i) specific uptake by the bacterium, ii) obstruction of movement of P through xylem due to attachment to biofilm structures, or iii) reduced uptake and transport of P through the xylem. A recent publication shows that genes involved in inorganic P uptake and transportation are repressed in grape during *X. fastidiosa* infection [41], which indicates that (iii) may explain the P deficiency observed here.

The concentrations of minerals in the tobacco examined in this study were within the range of adequate concentrations previously described for growth under field conditions [54], [55], though no data for greenhouse growth conditions were available. Assuming that field and greenhouse conditions are comparable, none of the minerals with reduced or increased concentrations during the *X. fastidiosa* infection process were modified drastically enough to constitute a deficit or excess for the normal growth of tobacco. However, disease symptoms do arise locally, and the average picture may not be completely indicative of the response at a cellular level.

With few exceptions, all three field hosts (grape, blueberry, and pecan) sampled showed increases in Ca and reductions in P when infected, with these changes more pronounced than in greenhouse experiments. A ∼40% reduction of P in infected leaves (calculated for pooled grape samples) indicates a deficiency that could impact normal plant growth in some environmental conditions. Greenhouse tobacco plants were fertilized and watered periodically, but field grown plants receive minimal, if any, fertilization or irrigation, so competition for nutrients between bacterium and plant host may be more pronounced and occur for longer periods of time, leading to greater differences. Unfortunately, due to the lack of replication from samples obtained from the same field and plant variety, extensive statistical analyses could not be conducted.

Ionomic characterization during plant infection is a useful approach to understand the impacts of pathogen infection on the host. For *X. fastidiosa*, this is particularly important because the bacterium-host molecular interactions are not completely understood. The findings presented here suggest that *X. fastidiosa* may use a common strategy of manipulating the host ionome directly or indirectly (as a plant response to infection) irrespective of the host plant species. This information will be valuable in the design of disease management strategies, since control of plant diseases by managing nutritional status of the host has been successful in the past [56], [57]. The information presented here will also inform future molecular studies on the interactions between the bacterium and the host regarding competition for specific mineral nutrients.
